# BRAF Inhibitors: Molecular Targeting and Immunomodulatory Actions

**DOI:** 10.3390/cancers12071823

**Published:** 2020-07-07

**Authors:** Ilaria Proietti, Nevena Skroza, Simone Michelini, Alessandra Mambrin, Veronica Balduzzi, Nicoletta Bernardini, Anna Marchesiello, Ersilia Tolino, Salvatore Volpe, Patrizia Maddalena, Marco Di Fraia, Giorgio Mangino, Giovanna Romeo, Concetta Potenza

**Affiliations:** 1 Department of Medical-Surgical Sciences and Biotechnologies, Dermatology Unit “Daniele Innocenzi”, Sapienza University of Rome, Polo Pontino, 04100 Latina, Italy; nevena.skroza@uniroma1.it (N.S.); simo.mik@hotmail.it (S.M.); mambrinalessandra@gmail.com (A.M.); veronica.balduzzi@gmail.com (V.B.); nicoletta.bernardini@libero.it (N.B.); anna.marchesiello90@gmail.com (A.M.); ersiliatolino@gmail.com (E.T.); salvatore.volpe@uniroma1.it (S.V.); patrizia.maddalena@uniroma1.it (P.M.); 16191@gmail.com (M.D.F.); concetta.potenza@uniroma1.it (C.P.); 2 Department of Medico-Surgical Sciences and Biotechnologies, Sapienza University of Rome, 04100 Latina, Italy; giorgio.mangino@uniroma1.it (G.M.); giovanna.romeo@uniroma1.it (G.R.); 3 Institute of Molecular Biology and Pathology, Consiglio Nazionale delle Ricerche, 00100 Rome, Italy

**Keywords:** BRAF-mutant melanoma, BRAF inhibitor, mechanism of action, melanoma, targeted therapy, tumour microenvironment

## Abstract

The BRAF inhibitors vemurafenib, dabrafenib and encorafenib are used in the treatment of patients with BRAF-mutant melanoma. They selectively target BRAF kinase and thus interfere with the mitogen-activated protein kinase (MAPK) signalling pathway that regulates the proliferation and survival of melanoma cells. In addition to their molecularly targeted activity, BRAF inhibitors have immunomodulatory effects. The MAPK pathway is involved in T-cell receptor signalling, and interference in the pathway by BRAF inhibitors has beneficial effects on the tumour microenvironment and anti-tumour immune response in BRAF-mutant melanoma, including increased immune-stimulatory cytokine levels, decreased immunosuppressive cytokine levels, enhanced melanoma differentiation antigen expression and presentation of tumour antigens by HLA 1, and increased intra-tumoral T-cell infiltration and activity. These effects promote recognition of the tumour by the immune system and enhance anti-tumour T-cell responses. Combining BRAF inhibitors with MEK inhibitors provides more complete blockade of the MAPK pathway. The immunomodulatory effects of BRAF inhibition alone or in combination with MEK inhibition provide a rationale for combining these targeted therapies with immune checkpoint inhibitors. Available data support the synergy between these treatment approaches, indicating such combinations provide an additional beneficial effect on the tumour microenvironment and immune response in BRAF-mutant melanoma.

## 1. Introduction

Melanoma is the deadliest form of skin cancer and results from the uncontrolled division of melanocytes. This is highlighted by the fact that melanoma accounts for only 4 percent of all dermatological cancers, but it is responsible for 80 percent of deaths [1]. Globally, over the last 50 years there has been a steady increase in the prevalence of melanoma with nearly 200,000 new cases diagnosed annually, with the highest rates recorded in Australia and New Zealand [2]. This has been paralleled by an improvement in survival rates likely as a result of earlier and better diagnosis, and the introduction of modern therapies including immunotherapy and targeted therapy [1,2]. In the US melanoma is the fifth most common cancer in men and the seventh most common cancer in women, and over the last 20 years 5-year survival rates have increased from approximately 80% to >90% [1,2]. In Europe in 2018, melanoma of the skin was ranked as the seventh most prevalent cancer with 144,209 new cases and 27,147 deaths [3]. In a previous report it was noted that there was a wide variation in incidence between countries, ranging from 2.2 in Greece to 19.2 in Switzerland (per 100,000 person years). The highest incidences tended to be recorded in Northern European countries (Estonia, Latvia, Lithuania, Scandinavia, the UK, and Ireland) and Western European (Austria, Belgium, France (metropolitan), Germany, Luxenberg, Switzerland and the Netherlands) [4].

Melanoma occurs as consequence of genetic mutations and environmental factors. Gene mutation lead to cell proliferation and growth, and the development of an invasive cell phenotype. Key risk factors predisposing to cutaneous melanoma include family history, male gender, older age, racial skin phenotype (Caucasian race increases risk), lighter coloured eyes (blue and hazel eyes have a greater risk than dark brown eyes), the presence of multiple dysplastic nevi, immunosuppression, sun sensitivity, and exposure to ultraviolet (UV) radiation in sunlight [1,2]. Two high risk groups are fair skinned individuals with red or blond hair with many freckles, and persons with darker hair and skin with high melanocytic nevi numbers [2]. Prevention (avoiding exposure to harmful UV radiation) and early detection are key initiatives to reduce the risk of developing a melanoma.

In recent years, the treatment of patients with melanoma has been advanced by the development of molecular targeted therapies and immunotherapies. This is based upon the finding that carcinogenesis is dependent on genetic mutations that activate mitogenic signalling, and that established tumours usually remain dependent on these signalling pathways. Thus, in-depth knowledge of the melanocyte signalling cascade offers therapeutic potential in patients with melanoma [5].

Recent advances in molecular genetics have provided a better understanding of the genetic mutations underpinning the pathogenesis and proliferation of melanocytes [6]. The majority of these mutations affecting the mitogen-activated protein kinase (MAPK) pathway, which is involved in the regulation of cell growth and proliferation [7,8]. Most notably, mutations affecting BRAF are found in more than half of patients diagnosed with cutaneous melanoma [9]. In BRAF-mutated melanoma, BRAF kinase becomes hyperactivated, resulting in increased cell proliferation and survival [7,10]. A better knowledge of the genetic and epigenetic changes of BRAF mutations through gene sequencing, as well as a fuller understanding of the underlying pathogenicity of melanoma, should help direct future targeted treatments and the development of a personalised approach to patient management. At the present time, three selective BRAF kinase inhibitors have been approved for use in the treatment of BRAF-mutant melanoma, including vemurafenib, dabrafenib and encorafenib [8]. Other genetic drivers in the pathogenesis of melanoma include *NRAS* and *neurofibromatosis 1* (*NF1*) mutations [11], but these will not be discussed in detail in this article.

This review summarises the mechanisms underlying the efficacy of BRAF inhibitors in the treatment of BRAF-mutant melanoma, including their inhibitory effect on constitutively activated BRAF kinase and consequent interference with the MAPK pathway, and their immunomodulatory role in the tumour microenvironment, leading to enhanced tumour recognition by the immune system and anti-tumour T-cell responses.

## 2. MAPK Pathway in Melanoma

The MAPK/extracellular signal-related kinase (ERK) pathway, under normal physiological conditions, plays a key role in the regulation of fundamental cellular processes, including cell growth, development, division, transformation, proliferation, migration and death (apoptosis). This is achieved through a broad-spectrum of interactions involving mitogens, growth factors and cytokines [7,8]. MAPK signaling is initiated via cell surface tyrosine kinase receptors, and subsequent activation of RAS, a membrane-bound GTPase. Transduction of the extracellular signals to the intracellular environment occurs through a hierarchical cascade of phosphorylation reactions which lead to the activation of specific kinases [7,8]. In particular, RAF (rapidly accelerated fibrosarcoma) protein kinases [of which there are three isoforms (A,B,C); namely, ARAF, BRAF and CRAF] are involved in the phosphorylation and activation of the MAP/ERK 1 and 2 kinases (MEK 1 and 2), which in turn phosphorylate the substrates ERK 1 and 2 [8]. Activated ERK is responsible for the phosphorylation of a range of substrates that are involved in the regulation of the gene expression which is essential for tumour growth and cytoskeletal functioning. These include effects on metabolism differentiation, proliferation, senescence and, ultimately, cell death.

RAF kinases, which are encoded by the *Raf* gene, exhibit serine/threonine protein kinase activity, with BRAF having the strongest activity and ARAF the weakest [7]. Of these three kinases, BRAF has the highest mutation rate and this has been reported to be up to 90% in melanoma tumours [7]. Activation of RAF is regulated by RAS. Upon stimulation by upstream factors, inactive RAS-GDP is converted to active RAS-GTP in the plasma membrane, which results in translocation of RAF to the membrane and the formation of RAF–RAS-GTP complexes [12]. Bound RAF kinases are activated through priming, including phosphorylation of key residues and dimerization through the kinase domain. RAF kinase domains have inactive and active conformations; dimerization helps produce the active conformation of RAFs, including BRAF, in the normal physiological setting [12]. 

Over-activating mutations affecting the MAPK/ERK pathway can lead to uncontrolled cell growth and this has been associated with a number of different neoplasms, including involvement in the pathogenesis/progression of melanomas [8]. Mutations of BRAF are the most common mutations leading to overactivation of the MAPK pathway, and BRAF-activating mutations are found in more than half of cutaneous melanomas [8,13]. The most common is the V600E mutation which accounts for 80–90% of BRAF mutations in melanomas and is present in almost 60% of cutaneous cases but is present in only 5% of mucosal melanomas [8]. The second most common mutation involves V600K which has been reported in approximately 8% of melanomas [8,13]. The p.V600E mutation, which leads to substitution of valine by glutamic acid at amino acid position 600 in the BRAF protein has been reported to result from the transversion c.1799T > A in exon 15 [8,13]. This mutation increases BRAF kinase activity ~700-fold compared with wild type BRAF [14]. While the base changes involved in this V600E mutation are not typical of those associated with UV radiation, there is clear evidence that they might result from error-prone replication of UV-damaged DNA, possibly as a consequence of multiple acute episodes of sun exposure [15]. The mutated gene leads to the production of a constitutively activated BRAF protein that dysregulates downstream MAPK signalling, promoting cellular proliferation and inhibiting apoptosis [7,10]. Oncogenic BRAF mutations promote spontaneous BRAF activation by various means, such as enhancing RAS-GTP-associated dimerization, or causing BRAF dimerization in the absence of RAS-GTP [12]. The structural mechanism underlying BRAF activation caused by the common V600E mutation is not fully understood, but there is evidence that the V600E substitution may promote conformational changes in BRAF leading to dimerization-induced activation [12].

## 3. Tumour Microenvironment in Melanoma

A number of studies have demonstrated that alongside genetic mutations, alterations in the tumour microenvironment (characterized by increased levels of proteins able to favour tumour invasion and infiltration) are responsible for melanoma proliferation [11]. In this regard, matrix metalloproteinases (MMPs), particularly MMP-9 and MMP-2, play a key role. These MMPs increase the degradation of components of the extracellular matrix, thus favouring tumour cell infiltration. Melanomas are associated with numerous mutations involving genes controlling cellular processes such us, proliferation (BRAF, NRAS and NF1), growth and metabolism [phosphatase and tensin homolog (PTEN) and KIT proto-oncogene receptor tyrosine kinase (KIT)], resistance to apoptosis [tumour protein p53 (TP53)], cell cycle control [cyclin-dependent kinase inhibitor 2A (CDKN2A)] and replicative lifespan [telomerase reverse transcriptase (TERT)] [11]. These genetic alterations typically lead to the aberrant activation of two main signalling pathways in melanoma: the RAS/RAF/MEK/ERK signalling cascade [also known as the mitogen-activated protein kinase (MAPK) pathway] and the phosphoinositol-3-kinase (PI3K)/AKT pathway [11]. The MAPK pathway is involved in T-cell receptor signalling. BRAF-mutant tumours with constitutive upregulation of the MAPK pathway, such as melanoma, can induce immune-escape mechanisms that make them immunologically “cold” and able to evade T-cell immune responses [16,17]. The tumours employ various mechanisms affecting different stages of the cancer–immunity cycle.

BRAF-mutant tumours create an immunosuppressive microenvironment that prevents the presentation of tumour antigens by antigen-presenting cells (such as dendritic cells and macrophages), and the T-cell priming that follows [16,18,19]. For example, BRAF^V600E^ cells adversely affect the maturation of dendritic cells and reduce their ability to produce the cytokines necessary for T-cell activation and expansion [16,19,20]. Tumour T-cell infiltrates are reduced in BRAF-mutated melanomas [21]. 

BRAF-mutant melanoma can escape recognition by effector T cells; for example, through low expression of melanoma differentiation antigens and by down-regulating the expression of human leucocyte antigen (HLA) class I molecules on melanoma cell surfaces [16,22]. HLA 1 is necessary for the presentation of antigens for recognition by T cells and, therefore, by reducing its expression, BRAF-mutant melanoma cells can escape recognition. 

The microenvironment of BRAF-mutant melanomas can also inhibit effector T-cell functions; for example, by promoting the accumulation of regulatory T cells and myeloid-derived suppressor cells (MDSCs) [16,17,19,20]. The regulatory T cells, via cell to cell contact-dependent mechanisms and immunosuppressive cytokines (interleukin (IL)-6 and IL-10) limit responses to effector T cells [16,21]. MDSCs include a variety of cells of myeloid origin which are able to potently suppress T cells 

In summary, BRAF-mutant melanomas have a tumour microenvironment involving upregulation of the MAPK signalling pathway that creates a pro-tumorigenic environment and an ineffective anti-tumour immune response [16]. 

## 4. BRAF Inhibitors: Mechanism of Action

### 4.1. Kinase Repression

The BRAF inhibitors vemurafenib, dabrafenib and encorafenib are approved for use in the treatment of patients with BRAF^V600^-mutant advanced melanoma (Table 1) [23,24,25,26,27,28,29,30]. These drugs are orally available, small molecule, selective inhibitors of BRAF kinase. By inhibiting BRAF they interfere with the MAPK signalling pathway that regulates the proliferation and survival of melanoma cells. 

They exhibit high specificity for BRAF^V600^-mutant cell lines, with this specificity thought to be due to preferential inhibition of the active conformation of BRAF, achieved by competitive occupation of the ATP binding pocket which stabilizes the kinase in its active conformation [8].

Preclinical studies demonstrated that vemurafenib and dabrafenib provide potent selective inhibition of kinase activity in BRAF^V600^-mutant melanoma cell lines, blocking ERK phosphorylation and cellular proliferation, and inducing G1 cell-cycle arrest and apoptosis [23,24,25,26,29,30]. Vemurafenib has confirmed activity against V600E, V600D and V600R mutant cell lines [23] and dabrafenib against V600E, V600D, V600R and V600K [24,25]. Inhibition is not seen with either drug in cells expressing wild type BRAF or non-V600 mutations [23,25,29]. In contrast, encorafenib, which targets V600E and V600K mutants, also displays some inhibitory effect in wild type BRAF [8,27,28]. In xenograft models of BRAF^V600E^-expressing melanoma, administration of these agents inhibits tumour growth, and at higher doses induces tumour regression [23,24,26,28,29].

In BRAF wild type cells, vemurafenib, dabrafenib and encorafenib can cause RAS-dependent paradoxical activation of the MAPK pathway, especially in cells that have pre-existing RAS mutations [10,12,24,31]. It may also select for the survival of non-BRAF^V600^ cells, causing drug resistance [10]. Combining a BRAF inhibitor with a MEK inhibitor (which acts by inhibiting kinases further downstream of BRAF in the MAPK pathway) prevents some of this increased MAPK signalling and provides more potent and durable inhibition of ERK signalling [24,31,32]. Dual MAPK pathway inhibition is a standard treatment option for BRAF-mutated melanoma.

### 4.2. Immunomodulatory Actions (Tumour Microenvironment)

BRAF inhibitors can reverse some of the immunosuppressive effects associated with the BRAF-mutant tumour microenvironment (discussed in Section 3), augmenting the immune response and turning them back into immunologically “hot” tumours [16,17,21,33]. This is achieved via several different mechanisms.

Firstly, BRAF inhibitors help restore an immune-stimulatory microenvironment in BRAF-mutant melanomas [16,17,21,33]. This is done by enhancing the expression of immune-stimulatory molecules/cytokines [19,34], reducing the expression of immunosuppressant molecules/cytokines [20,35,36,37], and reducing the accumulation of regulatory immune cells (e.g., regulatory T cells and MDSCs) [11,13,19,20,34,35,36,37,38,39,40,41] (Table 2).

Secondly, BRAF inhibitors increase T-cell infiltration into the tumour microenvironment of BRAF-mutant melanomas [16,17,21]. Increased CD8+ T-cell infiltration after BRAF-inhibitor treatment has been demonstrated in animal models, as well as from biopsies from patients [37,38,42,43,44]. Concurrent administration of a BRAF inhibitor improves the activity and infiltration of melanoma-specific adoptive T cells [45]. There is evidence from in vitro and in vivo studies that the enhanced tumour infiltration by T cells might be due to a reduction in vascular endothelial growth factor (VEGF) expression during BRAF inhibitor administration [20,22,35,36,45]. High VEGF levels cause blood vessel abnormalities that can restrict the entry of drugs and immune cells into tumours. In addition to T cells, an increased number of natural killer cells have been demonstrated in tumour infiltrates in a mouse model of BRAF-mutant melanoma after treatment with a BRAF inhibitor [41].

Thirdly, BRAF inhibitors enhance the recognition of melanoma cells by the immune system and so reduce the likelihood of the tumour escaping recognition by T cells [16,17,21,33]. Evidence from in vitro and in vivo studies indicate that BRAF inhibitors do this by increasing the expression of melanoma differentiation antigens, such as melanoma antigen recognised by T cells (MART-1), glycoprotein 100 (gp100), tyrosinase-related protein-1 (TYRP-1), TYRP-2 and dopachrome tautomerase (DCT) on BRAF-mutant cells [37,46], and also by increasing the expression of HLA 1 on the surface of BRAF-mutant melanoma cells [22,35].

Finally, BRAF inhibitors can improve the activity of effector T cells [16,21,33]. Studies using BRAF-mutant cell lines have shown that BRAF inhibitors increase T-cell activity, as indicated by an increase in IFNγ release [19,46]. Analysis of biopsies from patients with BRAF-mutant melanoma has shown that BRAF inhibitor treatment increases expression of markers of T-cell cytotoxicity, such as perforin and granzyme B [37].

MEK inhibitors also display some immunomodulatory activity, including in BRAF wild type melanoma [16]. In line with the effects of BRAF inhibitor monotherapy discussed above, the combination of a BRAF inhibitor and a MEK inhibitor has been shown to increase immune-stimulatory molecules/cytokines [35], reduce immunosuppressive cytokines [35,36,37], reduce VEGF expression [36], increase T-cell infiltrates [37], increase HLA 1 [35] and melanoma antigen [37] expression, and increase markers of T-cell cytotoxicity [37] in BRAF-mutant melanoma. Combination BRAF inhibitor plus MEK inhibitor treatment has also been shown to promote the cleavage of gasdermin E and release of HMGB1, suggesting that it may in part regulate the tumour immune microenvironment through pyroptosis (an inflammatory type of programmed cell death) [47].

The ability of BRAF inhibitors (and MEK inhibitors) to modify the tumour microenvironment and enhance anti-tumour immune responses in BRAF-mutated melanoma supports the idea that a combination of targeted therapy and immunotherapy could provide greater anti-tumour activity. Examples of immunotherapies used in melanoma include adoptive immunotherapy (passive transfer of activated T cells) and immune checkpoint inhibitors (which prevent the activation of T cells) [48]. Checkpoint inhibitors include monoclonal antibodies directed against programmed death receptor-1 (PD1), PD ligand-1 (PDL1), or cytotoxic T-lymphocyte-associated protein 4 (CTLA-4). These molecules are negative regulators of T-cell activity, and blocking their actions strengthens effector T cell functioning and anti-tumour responses [48]. Analysis of sequential biopsies from a patient with melanoma found a transient increase in T-cell infiltrate (followed by a decrease) during BRAF inhibitor monotherapy, which increased again and persisted after a dose of anti-CTLA4 antibody [42,43]. The combination of a BRAF inhibitor with either an anti-PD1 or anti-PDL1 checkpoint inhibitor has been shown to increase T-cell infiltration, the ratio of CD8+ T cells to regulatory T cells, and T-cell activity, compared with either agent alone in a murine model of BRAF-mutant melanoma [42,43]. Triple combination therapy with a BRAF inhibitor, a MEK inhibitor and pmel-1 adoptive cell transfer immunotherapy has been shown to increase T-cell infiltration into tumours and improve cytotoxicity in a murine model [49]. Studies in mice have also found that triple combination therapy with a BRAF inhibitor, MEK inhibitor and an anti-PD1 antibody produces greater anti-tumour activity than anti-PD1 monotherapy or any double combination amongst these therapies [49,50,51]. This triple combination therapy is associated with increased CD8+ T-cell infiltration, CD4+ cells and tumour-associated macrophages compared with anti-PD1 monotherapy [51]. Overall, these data suggest that the combination of targeted therapy and immunotherapy has a beneficial effect on the tumour microenvironment and T-cell response.

## 5. Clinical Implications

Most patients with newly diagnosed melanoma have early stage disease and surgical excision is the treatment of choice and is usually curative. This highlights the importance of regular skin checks and early detection. Some of these patients will relapse during the course of the disease, and others (about 10%) will present with advanced unresectable disease which may have already metastasized [11]. Medical treatment in these cases has been revolutionised in the last decade with the availability of several new therapies such as BRAF and MEK inhibitors and anti-PD1 and anti-CTLA4 immunotherapies. As a consequence of these developments, these newer therapies have become the mainstay of advanced melanoma therapy, and chemotherapy is now considered second-line at best [11].

BRAF inhibitors have been shown to rapidly suppress melanoma growth and control the malignancy in a large proportion of patients [11,52,53]. Although BRAF inhibitors can induce good responses in many patients with BRAF-mutant melanoma, in some cases, a reduction in effectiveness can be observed after 7–8 months of therapy [32,54,55]. Several mechanisms account for the reduction in tumour response to BRAF inhibitor therapy [52,53]. These include both primary (intrinsic) and secondary (acquired) mechanisms: primary applies to patients who do not respond to BRAF inhibitor therapy from the outset (approximately 15% of patients) [52]; whereas secondary mechanisms involve individuals who initially responded to BRAF inhibitor therapy, but subsequently relapsed [52]. Various pathways/mechanisms have been shown to potentially be involved in the development of acquired resistance [52]:

Activation of the PI3K-Akt pathway by upregulation of specific receptor tyrosine kinases (including insulin growth factor receptor 1 and platelet derived growth factor receptor β) in a non-ERK dependent manner, as well as induction of the pathway by epigenetically changed epidermal growth factor (EGFR). This dual activation of the P13K-Akt pathway promotes resistance, cell survival and proliferation

Activation of the MAPK pathway as a result of NRAS activating mutations, changes leading to maintenance of RAF dimerization, the ’BRAF inhibitor paradox’ (in which the BRAF inhibitor blocks MAPK signaling in mutant cells, but activates the MAPK pathway in non-mutant cells), and resistance to RAF inhibition through activation of HGF and its receptor MET (leading to the reactivation of the MAPK and P13K-Akt pathways)

Secondary mutations in MEK1 and 2 have both been linked to acquired resistance

The AXL receptor tyrosine kinase has been shown to be overexpressed in patients who have relapsed after treatment with BRAF and MEK inhibitors.

Combining a BRAF inhibitor with a MEK inhibitor (which acts further downstream on the MAPK pathway) can delay the development of resistance compared with BRAF inhibitor monotherapy and provides improved response and survival outcomes [32,56,57,58,59,60,61]. Consequently, dual MAPK inhibition with the combination of a BRAF inhibitor plus an MEK inhibitor is a standard-of-care approach for patients with unresectable or metastatic BRAF-mutant melanoma [62]. Approved combinations for patients with unresectable or metastatic melanoma include darafenib/trametinib, vemurafenib/cobimetinib and encorafenib/binimetinib [63]. Combination targeted therapy can also be beneficial in an adjuvant setting [64], and dabrefenib/trametinib has been approved for use as adjuvant treatment after complete resection of stage III melanoma [65]. However, in many patients, melanoma progression eventually occurs and resistance to treatment develops.

This led to an interest in immunotherapeutic agents to extend the therapeutic effect and induce long-acting anti-melanoma effects, and immune checkpoint inhibitors are another important treatment option for patients with advanced melanoma [11,16,52]. It has been reported that single-agent checkpoint inhibitors produced a clear clinical benefit over chemotherapy in metastatic melanoma, and these benefits appeared to be consistent across patient subgroups [66]. Moreover, combination of nivolumab plus ipilimumab, an anti- CTLA-4, was significantly more effective in term of objective response rate, progression-free survival and overall survival relative to ipilimumab used alone [66]. However, because checkpoint receptors play important roles in regulating autoimmunity, the major toxicities associated with the use of these drugs include autoimmune symptoms. The incidence of immune-related adverse events is relatively high, varying from 70% in patients treated with anti-PD-1/anti-PD-L1 antibodies to 90% in patients treated with anti-CTLA-4 [67]. Therefore, it is important to assess which patients we are dealing with and how they will respond to the therapy. Despite the noteworthy successes of immune check point blockade, to date, only a subset of patients achieve durable clinical responses [68]. Indeed, more than half of patients treated with anti-PD1 therapy fail to respond or eventually develop progression [69]. Some of this resistance may be due to immune mechanisms and, given that BRAF inhibitors (and MEK inhibitors) have favourable effects on antitumor immunity, switching to combination targeted therapy could help overcome some anti-PD1 resistance mechanisms. Clinical trials to date have demonstrated variable results with regard to efficacy, but BRAF/MEK-targeted therapy can be considered in patients with BRAF-mutant melanoma who do not respond to anti-PD1 therapy [69].

The safety and tolerability of treatment is an important clinical consideration. Paradoxical activation of the MAPK pathway by BRAF inhibitors can increase the risk of the other cutaneous malignancies developing [31]. Combination therapy with a BRAF inhibitor plus a MEK inhibitor is associated with reduced dermatological toxicity, although a slightly worse gastrointestinal adverse event profile, compared with monotherapy [70]. Combination therapy with BRAF inhibitors and some immunotherapeutic agents can also be associated with increased toxicity; in particular, the combination of vemurafenib and ipilimumab (an anti-CTLA4 monoclonal antibody) was associated with marked hepatotoxicity [21,48]. In contrast, the combination of a BRAF inhibitor with an anti-PDL1 antibody appears to be well tolerated [21,48].

Looking ahead, clinical trials evaluating the combination of a BRAF inhibitor (with or without a MEK inhibitor) and immunotherapy are ongoing [21,48]. Given that some studies found promising clinical responses but a problem with toxicity with certain combinations [71], other trials are evaluating sequential treatment with targeted therapy and immunotherapy in patients with BRAF-mutant melanoma, in order to identify the optimal sequencing and timing of treatment [21,48].

## 6. Conclusions

Advances in the treatment of metastatic melanoma have expanded over the last decade to include approaches such as targeted molecular therapy and immunotherapy, including T-cell checkpoint inhibition. These have been driven by the development of resistance to one or more of these therapeutic methods. The BRAF inhibitors vemurafenib, dabrafenib and encorafenib are used in the treatment of patients with BRAF^V600^-mutant advanced melanoma. They selectively target BRAF kinase and thus interfere with the MAPK signalling pathway that regulates the proliferation and survival of melanoma cells.

In addition to their molecularly targeted activity, BRAF inhibitors also exhibit immunomodulatory effects. The MAPK pathway is involved in T-cell receptor signalling, and interference with the pathway by BRAF inhibitors has beneficial effects on the tumour microenvironment and anti-tumour immune response in BRAF-mutant melanoma. This is achieved through several different mechanisms including: increasing immune-stimulatory cytokine levels; decreasing immunosuppressive cytokine levels; enhancing melanoma differentiation antigen expression and presentation of tumour antigens by HLA 1; and increasing intra-tumoral T-cell infiltration and activity. Overall, these effects promote recognition of the tumour by the immune system and enhance anti-tumour T-cell responses in BRAF-mutant melanoma.

Combining BRAF inhibitors with MEK inhibitors provides more complete blockade of the MAPK pathway. Moreover, the immunomodulatory effects of BRAF inhibition alone or in combination with MEK inhibition provide a rationale for combining these targeted therapies with immune checkpoint inhibitors. Available preclinical data support the synergy between these treatment approaches, indicating such combinations provide an additional beneficial effect on the tumour microenvironment and immune response in BRAF-mutant melanoma. Clinical trials are evaluating whether this translates into an improved clinical response and duration of response in patients. Areas for future research to address specific issues with the goal of improving treatment outcomes include:

Gaining a better understanding of resistance mechanisms for all potential therapies, through clinical trials and parallel translational/preclinical studies

Increasing the focus on personalised approaches to patient management, so as to maximise the benefits of available therapies whilst avoiding unnecessary toxicity

Studies to help define which treatment to start with, optimal dosage regimens/schedules, and when to consider adding another therapy

Identification of appropriate biomarkers to help physicians make more reliable predictions of likely response and possible toxicity.

## Figures and Tables

**Table 1:** BRAF Inhibitors [8,23,24,25,26,27,28,29,30].

INN	Chemical Name	Activity	FDA/EMA Approved Indications/Date of First Approval
**Vemurafenib**	*N*-[3-[5-(4-chlorophenyl)-1*H*-pyrrolo[2,3-b]pyridine-3-carbonyl]-2,4-difluorophenyl]propane-1-sulfonamide entry 1	Selectively binds to the ATP-binding site of BRAF kinase and inhibits its activity. Activity against BRAF^V600E, V600D, V600R^ mutant cell lines [23].	Monotherapy in adults with BRAF^V600^-mutation-positive unresectable or metastatic melanoma. FDA 2011 / EMA 2012
**Dabrafenib**	*N* -[3-[5-(2-aminopyrimidin-4-yl)-2-*tert*-butyl-1,3-thiazol-4-yl]-2-fluorophenyl]-2,6-difluorobenzenesulfonamide	Selectively binds to and inhibits the activity of BRAF. Activity against BRAF^V600E, V600D, V600R, V600K^ mutant cell lines [24,25,26].	Monotherapy in adults with unresectable or metastatic melanoma with BRAF^V600E^ mutation (FDA) or BRAF^V600^ mutations (EMA). Combination therapy with trametinib in patients with unresectable or metastatic melanoma with BRAF^V600E^ or BRAF^V600K^ mutations (FDA) or BRAF^V600^ mutations (EMA), and also adjuvant treatment of adults with stage III melanoma with a BRAF^V600^ mutation, following complete resection (EMA). FDA 2013 / EMA 2013
**Encorafenib**	Methyl *N*-[(2*S*)-1-[[4-[3-[5-chloro-2-fluoro-3-(methanesulfonamido)phenyl]-1-propan-2-ylpyrazol-4-yl]pyrimidin-2-yl]amino]propan-2-yl]carbamate	Selective ATP-competitive RAF kinase inhibitor. Activity against BRAF ^V600E, V600D, V600K^ mutant cell lines [8,27,28]	Combination therapy with binimetinib in adults with unresectable or metastatic melanoma with a BRAF^V600^ mutation (EMA) or BRAF^V600E^ or BRAF^V600K^ mutation detected by FDA-approved test (FDA). FDA 2018 / EMA 2018

EMA = European Medicines Agency; FDA = US Food and Drug Administration.

**Table 2:** Mechanisms by which BRAF Inhibitors Help Restore an Immune-stimulatory Tumour Microenvironment in BRAF-mutant Melanoma [11,13,18,20,34,35,36,37,38,39,40,41].

Effect	Findings
Increased expression of immune-stimulatory molecules/cytokines	Increased CD40L and IFNγ expression on intratumoural CD4+ TILs in a murine model of melanoma [19] Increased IL-12 and TNFα production and surface marker expression (CD80, CD83, CD86) in DCs co-cultured with BRAF-mutant melanoma cells [20,34] Increased levels of IFNγ, TNFα and the chemokine CCL4 in melanoma patient serum samples [38]
Reduced expression of immunosuppressive molecules/cytokines	Decreased expression of IL-1, IL-6, IL-8, IL-10, VEGF in BRAF-mutant melanoma cells [20,35,36] Decreased expression of IL-6 and IL-8 in melanoma patient biopsies [37] Decreased levels of IL8 (CXCL8) in melanoma patient serum samples [38]
Reduced accumulation of regulatory immune cells and regulatory chemokines	Reduced accumulation of Tregs and MDSCs in murine models of melanoma [19,40] Decreased MDSC level in melanoma patient blood samples [39] Decreased expression of CCL2, increased ratio of CD8+ T cells to Tregs in a murine BRAF-mutant melanoma model [41]

CCL2 = CC-chemokine ligand 2; DC = dendritic cell; IFN = interferon; IL = interleukin; MDSC = myeloid-derived suppressor cell; NK = natural killer; TIL = tumour-infiltrating cell; Treg = regulatory T cell; VEGF = vascular endothelial growth factor.
